# Material-dependent oxygen transport governs ischemia-reperfusion injury and drug response in a kidney microphysiological system

**DOI:** 10.3389/ftox.2026.1854389

**Published:** 2026-07-10

**Authors:** Naokata Kutsuzawa, Hiroko Nakamura, Kenta Shinha, Hiroshi Kimura

**Affiliations:** 1 Micro/Nano Technology Center, Tokai University, Hiratsuka, Kanagawa, Japan; 2 Division of Pulmonary Medicine, Department of Medicine, Tokai University School of Medicine, Isehara, Kanagawa, Japan

**Keywords:** hypoxia, ischemia reperfusion injury, microphysiological system, oxygen consumption rate (OCR), oxygen transport, PDMS, PET

## Abstract

Oxygen availability is a critical yet often overlooked factor in cell culture, and discrepancies between nominal oxygen settings and the actual cellular microenvironment can compromise model validity. This limitation is particularly relevant in modeling ischemia–reperfusion (I/R) injury, where dynamic changes in oxygen supply critically influence cellular responses. In particular, the role of oxygen transport in microphysiological systems and conventional culture platforms remains insufficiently understood, limiting the reproducibility of disease models and the interpretation of drug responses. In this study, we systematically evaluated oxygen supply and cellular responses in three culture platforms: cell culture inserts, polydimethylsiloxane (polydimethylsiloxane)-based microfluidic chips, and polyethylene terephthalate (polyethylene terephthalate)-based microfluidic chips with identical geometries but different oxygen permeabilities. Oxygen distribution and oxygen consumption rate (OCR) were estimated using finite element method (FEM) simulations, and the resulting predictions were interpreted in the context of experimental observations obtained with RPTEC/TERT1 cells under I/R conditions. Cellular responses were assessed by morphology, viability, and gene expression analysis, and pharmacological responses were evaluated using the renoprotective compound CDDO. finite element method analysis revealed that oxygen availability at the cell surface varied markedly among platforms despite identical nominal oxygen conditions. PDMS chips maintained high oxygen levels due to high gas permeability, preventing the establishment of oxygen-limited conditions, whereas PET chips and inserts exhibited significant oxygen depletion. These differences were consistent with experimental observations: PDMS chips failed to induce I/R-associated cellular injury, whereas PET chips and inserts enabled hypoxia-induced responses. Furthermore, drug responses varied across platforms, with CDDO exhibiting distinct efficacy profiles depending on the severity of oxygen limitation. These findings indicate that oxygen transport, rather than nominal oxygen settings, is a major determinant of cellular phenotype and drug response *in vitro*. This study provides a framework for designing physiologically relevant cell culture systems based on oxygen transport principles and highlights the importance of integrating material properties, device design, and culture conditions to achieve accurate and reproducible biological outcomes.

## 1 Introduction

Renal ischemia-reperfusion (I/R) injury is one of the primary mechanisms underlying acute kidney injury (AKI) (Rewa and Bagshaw, 2014; Ronco et al., 2019), which is a syndrome characterized by an abrupt decline in kidney function, accompanied by high mortality, impaired organ function, and the onset of chronic kidney disease (Hobson et al., 2015). Renal ischemia is an unavoidable condition that occurs in a variety of clinical situations, including kidney transplantation, cardiovascular surgery, and circulatory failure. During the ischemic phase, the cessation of oxygen and nutrient supply significantly impairs renal metabolism, and rapid reperfusion during the reperfusion phase leads to oxidative stress, mitochondrial dysfunction, and cell death, thereby impairing renal function (Lasorsa et al., 2024; Pefanis et al., 2019). Quantitatively, in the human kidney, the cortical O_2_ partial pressure is approximately 50 mmHg under physiological conditions and falls to ∼5–25 mmHg in the outer medulla, whereas during ischemia, tissue PO_2_ can fall below ∼5 mmHg (Evans et al., 2008; Hansell et al., 2013). Upon reperfusion, abrupt reoxygenation triggers a burst of mitochondrial superoxide production and a transient increase in tissue ROS (Pefanis et al., 2019; Huang et al., 2024; Lasorsa et al., 2024). *In vitro* studies on human RPTECs further show that hypoxia–reoxygenation perturbs the intra- and extracellular metabolome and modulates transporter transcription within hours of injury (Faucher et al., 2023). I/R injury also affects renal structures in a heterogeneous manner. The S3 segment of the proximal tubule, located in the outer stripe of the outer medulla, is the most consistently injured region because of its high oxidative metabolism, low glycolytic reserve and dependence on Na^+^/K^+^-ATPase under conditions of borderline O_2_ supply (Evans et al., 2008; Pefanis et al., 2019). The glomerulus is in general less acutely vulnerable, although glomerular endothelial dysfunction is increasingly recognized as a contributor to peritubular capillary rarefaction and the chronic sequelae of AKI (Lasorsa et al., 2024). Furthermore, I/R injury accelerates the progression to chronic kidney disease (CKD) and, in patients already suffering from CKD, contributes to the progression to end-stage renal disease; its prevention and management are crucial. Therefore, establishing an experimental model that can reproducibly and quantitatively assess renal I/R injury is a critical challenge for both elucidating the pathophysiology and developing therapeutic agents (Malek and Nematbakhsh, 2015; Pefanis et al., 2019; Huang et al., 2024). Previous research on renal I/R injury has primarily used animal models (Skrypnyk et al., 2016). However, animal testing has limited predictive accuracy due to species differences between animals and humans, as well as ethical constraints, higher costs, and low throughput. Consequently, the use of *in vitro* models based on human-derived cells is garnering increasing attention. Unlike animal experiments, *in vitro* systems allow precise control of conditions such as oxygen levels and medium perfusion (Russ et al., 2007); however, conventional static culture systems and those using cell culture inserts cannot reproduce dynamic aspects such as fluid shear stress. For example, the axial fluid shear stress experienced by proximal tubule cells *in vivo* is estimated to be in the range of 0.1 to 1 dyne/cm^2^ (Essig et al., 2001; Duan et al., 2008; Maggiorani et al., 2015). Not only can this not be replicated under static culture conditions, but the reproduction of cellular structure and function is also incomplete (van Duinen et al., 2015; Wilmer et al., 2016). Furthermore, it is difficult to strictly control the changes in the oxygen environment associated with ischemia and reperfusion, and a physiologically valid renal I/R model has not yet been fully established (Shiva et al., 2020; Wang et al., 2024; Harwood et al., 2022; Smith et al., 2024).

In recent years, kidney models utilizing microphysiological systems (MPS) and organ-on-a-chip technology have been reported, and approaches to replicate perfusion culture, cell-cell interactions, and disease-related microenvironments are progressing (Nguyen et al., 2023; Kimura et al., 2023; Kimura et al., 2024; Kimura et al., 2025). Microfluidic platforms have been developed to recapitulate individual aspects of the renal microenvironment, including fluid shear stress alone (Jang et al., 2013; Homan et al., 2016; Kann et al., 2022), low O_2_ conditions alone or with integrated sensors (Brennan et al., 2014; Azimzadeh et al., 2022), and the combination of hypoxia with mechanical stimulation (Vormann et al., 2022; Shaughnessey et al., 2024; Izadifar et al., 2024). Recent reviews provide an up-to-date overview of kidney-on-chip technology and its applications to disease modeling and drug evaluation (Nguyen et al., 2023). In the context of renal I/R modeling, several on-chip systems have demonstrated that cellular injury can be induced under hypoxic and hyponutritional conditions, exacerbated by reperfusion, and attenuated by ischemic protective agents (Vormann et al., 2022; Chethikkattuveli Salih et al., 2022; Tool et al., 2024). Furthermore, high-throughput MPS studies have shown that the combination of low O_2_ conditions and perfusion stimulation is important for reproducing I/R-associated injury (Shaughnessey et al., 2024). These studies have substantially advanced *in vitro* renal I/R modeling by incorporating key biological and mechanical features of the renal microenvironment. However, many prior studies have primarily focused on oxygen settings, nutrient deprivation, flow, or shear stress, whereas the influence of device material properties on the actual cellular oxygen environment has not necessarily been examined in a systematic manner. This issue is particularly important because polydimethylsiloxane (PDMS), a widely used material for microfluidic chips, has high gas permeability (Banik et al., 2023). Therefore, even when a chip is placed under nominally low O_2_ conditions in an incubator, oxygen diffusion through PDMS may maintain a higher-than-expected oxygen concentration around the cells. Although such material-dependent oxygen transport could affect the reproducibility and interpretation of *in vitro* ischemia models, systematic validation using microfluidic chips with identical geometry but different gas permeability remains limited. In addition, when evaluating the utility of microfluidic chips, comparison with conventional static culture systems is essential. Cell culture inserts are widely used as standard platforms for epithelial cell culture; however, their oxygen-supply characteristics and the pericellular oxygen environment have not been sufficiently characterized. It also remains unclear to what extent the oxygen concentration set in the incubator reflects the actual cellular microenvironment in each culture platform. Therefore, quantitative evaluation of oxygen distribution and cellular oxygen consumption behavior is necessary for both microfluidic chips and conventional insert systems. Furthermore, limited information is available on how platform-dependent oxygenation affects not only the reproducibility of I/R injury but also the interpretation of drug responses. To our knowledge, no previous study has explicitly compared microfluidic chips with identical geometry but distinct gas permeability against a conventional insert reference under matched nominal O_2_ settings to dissect the contribution of material-dependent oxygen transport to cellular responses. This gap defines the focus of the present study.

In this research, we aimed to clarify the importance of the oxygen supply function in the development of a renal I/R model and conducted a comparative study using double-layered microfluidic chips of the same geometry, each composed of material with different oxygen permeability. We used finite element method (FEM) simulations to compare oxygen supply under culture conditions for low-oxygen-permeable polyethylene terephthalate (PET) chips, high-oxygen-permeable PDMS chips, and conventional cell culture inserts, thereby confirming their differences. Subsequently, we evaluated cell morphology and viability under I/R conditions using the human proximal tubule epithelial cell line RPTEC/TERT1. Furthermore, to verify the suitability of this model as an MPS, we conducted drug tests with 2-cyano-3,12-dioxo-olean-1,9-dien-28-oic acid (CDDO), an ischemic protective agent. Through this series of investigations, we suggest that in developing MPS models, including the renal I/R model, it is essential to comprehensively optimize the oxygen-supply characteristics of the device materials and the corresponding culture conditions.

## 2 Materials and methods

### 2.1 Microfluidic chip and experimental setup

To develop a renal I/R model, we employed a double-layered microfluidic chip with a microporous membrane. The chip structure consists of top and bottom flow channels separated by a microporous membrane, enabling cell culture and controlled mass transport under perfusion conditions (Kimura et al., 2024). To investigate the effects of oxygen permeability, we prepared two types of double-layered microfluidic chips made from different materials. Specifically, we compared a microfluidic chip made of PET, which has low-oxygen permeability, with one made of PDMS, which has high oxygen permeability.

**FIGURE 1:**
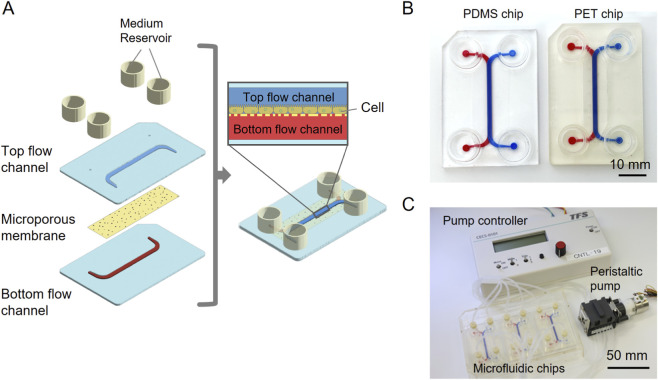
Double-layered microfluidic chips and perfusion system. **(A)** Schematic illustration of the double-layered microfluidic chip. The chip consists of two microchannels separated by a microporous membrane, on which cells are cultured as a confluent monolayer. **(B)** Photographs of the microfluidic chips fabricated from different materials: PDMS (left) and PET (right). **(C)** Photograph of the perfusion culture system. Three chips are mounted on a single plate, and each medium reservoir of inlet and outlet is connected to a peristaltic pump via silicone tubing.

We employed Fluid3D-X®, a product of Tokyo Ohka Kogyo (Kanagawa, Japan), as the PET chip (Imaoka et al., 2024; Kutsuzawa et al., 2025a; Kutsuzawa et al., 2025b). The PET chip has a typical double-layered microchannel structure, separated by a microporous membrane with a pore size of 0.45 µm and medium reservoirs in each port (Figure 1A). The microfluidic channel in the cell culture section of the PET chip has a width of 2 mm, a height of 0.7 mm, a length of 32 mm, and an effective culture area of 64 mm^2^. A PDMS microfluidic chip with the same geometry as the PET chip was also fabricated (Figure 1B). The PDMS chip was fabricated using the previously reported fabrication process (Kimura et al., 2008). Briefly, PDMS layers with microchannel structures were fabricated by soft lithography. Polymethyl methacrylate master molds for soft lithography were made by a 3D modeling machine (MODELA MDX-50, Roland, Shizuoka, Japan). A 10:1 mixture of unpolymerized PDMS (SILPOT184; Dow Toray, Tokyo, Japan) and catalyst was poured into the master mold and baked in an oven at 75 °C for 2 h. For permanent bonding to the PDMS layers, the surface of a microporous membrane was coated with a solution of 6.7% (v/v) 3-aminopropyltriethoxysilane (KBE-903, Shin-Etsu Chemical, Tokyo, Japan) and 2.5% (v/v) glutaraldehyde (17026-32, Kanto Chemical, Tokyo, Japan). Holes (3 mm in diameter) were made in the PDMS layer on the top flow channel side using a biopsy punch (BPP-30F, Kai Medical, Gifu, Japan) at the inlet and outlet ports of the top and bottom flow channels. The PDMS chip was assembled by sandwiching the microporous membrane between the plasma-treated PDMS layers.

In both double-layered microfluidic chips, continuous perfusion culture was achieved using a commercially available peristaltic pump (AQ-RP6R-001, Takasago Fluidic Systems, Nagoya, Japan) and 600 mm silicone tubes (Figure 1C). After initial calibration in accordance with the manufacturer’s instruction manual, the medium flow rate can be adjusted between 3.8 μL/min and 380 μL/min using the designated controller. We previously conducted experimental tests to verify the reliability of this pump’s flow rate and confirmed that it falls within the manufacturer’s guaranteed tolerance range of ±10%. The reservoir port on the chip is not tightly closed but loosely covered by the plate lid, allowing the culture medium surface inside the reservoir to exchange gases with the incubator atmosphere.

### 2.2 FEM simulation

The oxygen concentration in the vicinity of the cells and the oxygen consumption rate (OCR) under each cell culture condition in each cell culture system were evaluated using the finite element method (FEM) implemented in COMSOL Multiphysics (version 6.4, COMSOL, Burlington, MA, United States). In the COMSOL model, the culture medium, microporous membrane, and cell layer were constructed for both the insert and the microfluidic chips (Supplementary Figure S1).

Oxygen was supplied to the medium from the air–liquid interface at the open reservoir ports, which were not sealed and therefore exchanged gas with the surrounding incubator atmosphere and, in the PDMS chip only, also through the gas-permeable channel walls. In the PET chip, the channel walls are effectively impermeable to O_2_ over the experimental timescale, so the reservoir surface is the sole O_2_ source. Oxygen supply was modeled by imposing a fixed oxygen concentration at the corresponding interfaces. In the control (in-air) condition for the microfluidic chips, culture medium equilibrated with in-air oxygen was supplied from the inlet reservoir at a flow rate of 130 μL/min. This assumption was based on FEM simulation results. The simulation suggested that the medium approached a near-saturated dissolved oxygen concentration corresponding to the gas-phase oxygen concentration after passing through 200 mm of silicone tubing, owing to the tubing’s high gas permeability (Supplementary Figure S2). The total length of the silicone tube used in the experiment was three times this distance (600 mm), and it is estimated that in the actual experimental setup, the medium passing through the tube reached nearly saturated dissolved oxygen concentration before entering the chip.

The initial oxygen concentration and the interface concentration were set to the dissolved oxygen concentration (Supplementary Table S1). The oxygen levels for the ischemic conditions (5% O_2_ and 1% O_2_) were set to 1/3.7 and 1/18.6 of the in-air oxygen concentration, respectively.

The cell layer was modeled as a 10 μm‐thick monolayer. The cellular OCR (mol/m3/s) was defined using the Michaelis‐Menten equation as follows (Equation 1) (Rogers et al., 2024).

$$OCR=\frac{{OCR}_{Basal}\times C_{\left(t\right)}}{K_{m}+C_{\left(t\right)}}$$ (1)

Here, *C*_(*t*)_ represents the oxygen concentration in the cell layer at time *t*, and *K*_*m*_ denotes the Michaelis constant. OCR_Basal_ represents the baseline oxygen consumption rate. When *C*(*t*) ≤ 0, the OCR was set to 0 mol/m^3^/s.

### 2.3 Cell culture

Human telomerase reverse transcriptase immortalized RPTEC/TERT1 cells were obtained from the American Type Culture Collection (CRL-4031, ATCC, Manassas, VA, United States) and cultured in a hormonally defined, serum-free medium consisting of Dulbecco’s modified Eagle’s Medium F12 (30-2006, ATCC, Manassas, VA, United States) supplemented with a growth kit (ACS-4007, ATCC, Manassas, VA, United States) and 0.1 mg/mL G418 sulfate (10131035; Thermo Fisher Scientific, Waltham, MA, United States) at 37 °C and 5% CO_2_. RPTEC/TERT1 was chosen because it is the most extensively characterized hTERT-immortalized human proximal-tubule epithelial cell line and retains key proximal-tubule features at a level close to the physiological levels — including megalin, γ-glutamyl transpeptidase, aminopeptidase N, ZO-1, polarized epithelial morphology and primary cilia — without inactivation of the p53 or pRB pathways (Wieser et al., 2008). Furthermore, it is well-suited for experiments involving inserts or MPS and is widely used in our laboratory for studies that model the kidney (Nihei et al., 2025).

The cells were cultured in tissue culture-treated dishes (100 mm in diameter) until the experiment. The culture medium was changed every other day, and the cells were passaged at 70% confluence. No more than six passages were used for culture experiments.

The PDMS chip was sterilized by UV irradiation (20 W, NB-5, Nichiban Co., Ltd.) for 20 min. The PET chips were supplied by the manufacturer after being sterilized by gamma irradiation. The microfluidic chips were coated with Laminin 511 (iMatrix-511 silk, 892021; MATRIXOME, Osaka, Japan) at 37 °C for an hour. Then, the cells were seeded at a density of 3.0 × 10^5^ cells/cm^2^ into the top flow channel inlets of each chip using a micropipette, and the bottom flow channel was filled with medium in the same manner. After seeding the cells, the microfluidic chips were put in a plate and incubated at 37 °C and 5% CO_2_ to allow the cells to attach to the membrane. After 3–5 h, when cell attachment was confirmed, 650 μL of medium was added to the medium reservoirs using a multi-channel pipette. After filling each reservoir with culture medium, we began perfusing at approximately 4 μL/min using the perfusion system. Pre-culture was conducted for up to 17 days, and the culture duration and medium perfusion rate were adjusted in stages for each experiment based on cell condition as observed under microscopic examination. This extended pre-culture period was selected to ensure complete polarization, the formation of mature tight junctions, and stable baseline barrier function in RPTEC/TERT1 cells; this approach was recommended in the initial characterization of this cell line (Wieser et al., 2008) and has also been adopted in subsequent proximal tubule chip studies (Vormann et al., 2022; Tool et al., 2024). The medium was changed every 2 or 3 days.

In addition, we employed cell culture inserts (3,460, Corning, NY, United States), which consist of polyethylene terephthalate (PET) track-etch membranes with 0.4 µm-diameter pores, placed at a density of 4 × 10^6^ pores/cm^2^, as a representative conventional culture system. The use of the same PET membrane in both the chips and the insert allows the cellular substrate composition to be matched between the two platforms; the difference between the two systems is therefore the surrounding material (chips vs. inserts) and the medium-handling regime (perfusion vs. static).

### 2.4 Protocol for simulating ischemia-reperfusion conditions

The ischemia-reperfusion experiment consisted of a pre-experiment period, an ischemia period, and a reperfusion period; during the pre-experiment and reperfusion periods, perfusion was maintained at 130 μL/min (approximately 0.1 dyn/cm^2^). During the ischemia period (24 h), the ischemia treatment groups were cultured statically in glucose-free culture media in a multi-gas incubator at the respective oxygen concentrations (5% O_2_ and 1% O_2_) with the lid placed on the plate. Subsequently, during the reperfusion period (24 h), the insert was replaced with a glucose-containing medium and cultured in an in-air incubator, while the microfluidic chips were perfused at 130 μL/min in an in-air incubator. The control group of the microfluidic chips was perfused with culture media with glucose at 130 μL/min during the ischemia period. All inserts were cultured under static conditions.

### 2.5 Quantitative real-time PCR

Total RNA from the cells in the inserts was collected using TRIzol (15596018, Thermo Fisher Scientific, Waltham, MA, United States) and isolated using Direct-zol RNA Microprep (R2062, Zymo Research, Irvine, CA, United States). After measuring the amount of RNA using a NanoDrop Lite spectrometer (ND-LITE; Thermo Fisher Scientific, Waltham, MA, United States), 1 µg of total RNA was reverse transcribed using the iScript cDNA Synthesis Kit (1,708,891, Bio-Rad, Hercules, CA, United States) according to the manufacturer’s instructions. Subsequently, complementary DNA was used for real-time PCR. Each sample was tested in at least two technical replicates. Gene expression levels of HIF1A, GLUT1, VEGFA, KIM-1, and HPRT1 were evaluated by quantitative real-time PCR using SsoAdvanced Universal SYBR Green Supermix (1725271, BioRad, Hercules, CA, United States). The primer sequences are shown in Supplementary Table S2. Gene expression levels normalized to HPRT1 were used as a reference, and the calculated fold difference was compared with those in the in-air cell control.

### 2.6 Viability assay

Cell viability was evaluated by both qualitative microscopic observation and quantitative analysis using a Cell Counting Kit-8 (CK04, Dojindo, Kumamoto, Japan). Cell morphology was observed by phase-contrast microscopy after the ischemic treatment and after the reperfusion period under each experimental condition.

For the insert experiments, after the reperfusion period, the culture medium from both the insert and the well was removed. A solution containing approximately 10% (v/v) Cell Counting Kit-8 reagent in pre-warmed culture medium was prepared, and 0.5 mL of this solution was added to the insert. After incubation at 37 °C under 5% CO_2_ for 30 min, the solution was collected, and the absorbance at 450 nm was measured using a microplate reader.

For the microfluidic chip experiments, after completion of the reperfusion period, the medium in the inlet and outlet reservoirs was removed using a micropipette. The medium in the bottom channel was also removed, leaving medium only in the top channel. Subsequently, 0.3 mL of Cell Counting Kit-8 solution (approximately 10% (v/v) in culture medium) was introduced into the inlet of the top channel. After incubation at 37 °C under 5% CO_2_ for 30 min, 120 µL of the solution was collected from the outlet and reintroduced into the inlet to mix the contents of the channel. This mixing procedure was repeated twice, after which the solution was collected from the outlet, and the absorbance at 450 nm was measured using a microplate reader.

The absorbance values were normalized to the control condition in each experiment, defined as 100%.

### 2.7 Statistical analysis

All data are presented as the mean values obtained from at least three independent experiments, with error bars representing the standard deviation (SD). For comparisons in the I/R modeling and Real-Time PCR experiments, one-way analysis of variance (ANOVA) was performed, followed by Tukey’s multiple comparison test. For the drug assay, one-way ANOVA was performed, followed by Dunnett’s test. Differences were considered statistically significant at *p* < 0.05. All statistical analyses were performed using GraphPad Prism (version 10.4.2, GraphPad Software, MA, United States).

## 3 Results and discussion

### 3.1 Oxygen supply and OCR simulation

**FIGURE 2:**
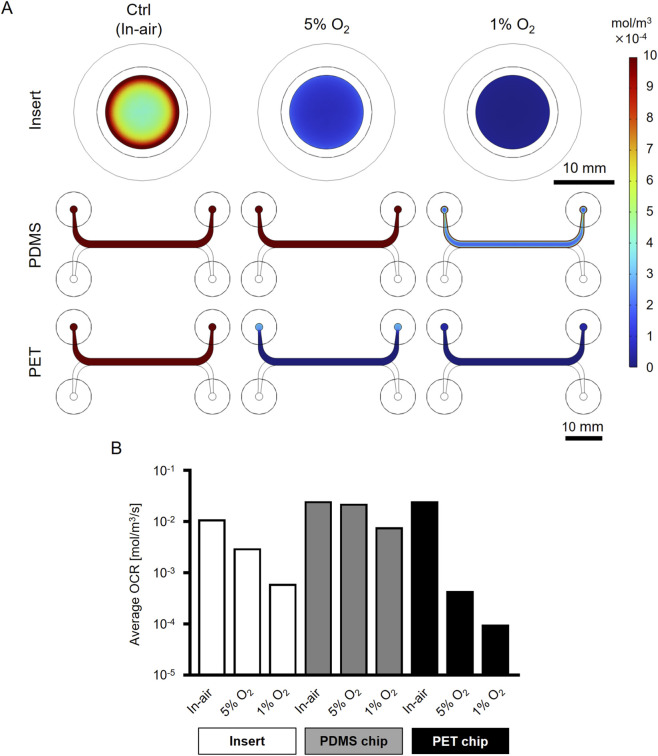
FEM-based simulation of oxygen distribution and oxygen consumption rate (OCR). **(A)** Simulated oxygen concentration at the apical surface of the cell layer after 24 h of culture under different oxygen conditions (in-air, 5% O_2_, and 1% O_2_) in the insert, PDMS chip, and PET chip. Microfluidic chips under the control (in-air) condition were perfused at a flow rate of 130 μL/min, whereas all other conditions were static. Severe oxygen depletion was predicted in the insert at 1% O_2_ and in the PET chip at both 5% and 1% O_2_. **(B)** Simulated 24 h-averaged OCR in the cell layer. Under the control condition, OCR in the microfluidic chips was comparable to basal OCR, whereas it was reduced in the insert. Under reduced-oxygen conditions, OCR markedly decreased in the insert and PET chip, indicating oxygen limitation.

FEM analysis revealed that oxygen transport within the culture systems varied markedly across platforms and material properties. Under the control (in-air) condition, the oxygen concentration at the apical surface of the cell layer was higher and more spatially uniform in the microfluidic chips (flow rate: 130 μL/min) than in the insert (static culture), regardless of the chip material (Figure 2A). In all culture systems, decreasing the oxygen level set in the incubator reduced the oxygen concentration at the cell surface; however, the extent of this reduction varied substantially among the platforms. Notably, in the insert under 1% O_2_ and in the PET chip under 5% and 1% O_2_, the oxygen concentration at the cell surface dropped below 1 × 10^−4^ mol/m^3^ after 24 h, indicating a severely oxygen-limited environment. In contrast, the PDMS chip maintained relatively high oxygen levels even under low-oxygen conditions.

Consistent with these findings, the 24 h-averaged OCR calculated using FEM varied with oxygen availability (Figure 2B). Under control (in-air) conditions, the OCR in microfluidic chips was comparable to the basal OCR, whereas in the insert, it was reduced to approximately half. Importantly, the PDMS chip maintained OCR levels comparable to control (in-air) even under 5% and 1% O_2_, indicating sufficient oxygen supply. In contrast, OCR in the insert under 1% O_2_ and the PET chip under 5% and 1% O_2_ decreased markedly under reduced-oxygen conditions, reaching levels as low as 1/40–1/250 of the basal OCR.

To evaluate the robustness of the FEM model, we conducted a sensitivity analysis focusing on OCR and *K*_*m*_, the dominant parameters governing oxygen consumption in the simulation. Based on values reported in the literature, we examined two extreme parameter combinations: the largest reported OCR with the smallest reported *K*_*m*_, and the smallest reported OCR with the largest reported *K*_*m*_. As shown in Supplementary Figure S3, although these parameter variations resulted in numerical differences in the predicted oxygen concentration and OCR, they did not alter the overall trends or the main conclusions of this study. These results support the robustness of the simulation model within the range of parameters examined and indicate that the main conclusions are not highly sensitive to plausible variations in OCR and *K*_*m*_.

Further analysis under static conditions (in-air, without medium perfusion) using microfluidic chips revealed distinct differences in oxygen supply mechanisms between materials. In the PDMS chip, sufficient oxygen levels were maintained even without perfusion, whereas in the PET chip, oxygen was depleted within the channel under static conditions (Supplementary Figure S4). These results indicate that oxygen supply in PDMS is strongly influenced by diffusion through the material, whereas in the PET chip, oxygen availability is more dependent on medium perfusion.

In addition, spatial analysis showed that oxygen concentration was lower toward the center of the culture area under static conditions in the insert (Figure 2A). This gradient is attributed to oxygen transport from the basal chamber through the microporous membrane, resulting in non-uniform oxygen distribution across the culture region (Supplementary Figure S5).

Taken together, these results suggest that oxygen transport is governed not only by incubator oxygen settings but also by material-dependent gas permeability and perfusion conditions. In particular, the high gas permeability of PDMS attenuates the establishment of severe oxygen consumption-limited conditions, whereas the PET chip and insert allow substantial oxygen limitation. Therefore, controlling incubator oxygen levels alone is insufficient, and platforms with limited oxygen permeability, such as PET chips or inserts, are more suitable for generating severe oxygen-limited conditions relevant to *in vitro* ischemia modeling.

Although it could not be implemented in this study, direct in-chip oxygen measurement is now a rapidly maturing area: recent reviews summarize the principal optical (luminescence-lifetime optodes based on Pt/Pd-porphyrin or [Ru (dpp)_3_]^2+^ indicators; sensor spots; nano-enabled and ratiometric variants) and electrochemical (Clark-type and microelectrode array) strategies for in-chip O_2_ measurement (Azimzadeh et al., 2022; Ling et al., 2026). Multifunctional sensor-integrated organ chips have recently demonstrated continuous, label-free on-chip monitoring of O_2_, trans-epithelial electrical resistance (TEER) and pH at physiologically relevant oxygen tensions (Izadifar et al., 2024). Most pertinent to the present work, Kann et al. integrated optical O_2_ optodes with a membrane-bilayer human renal proximal-tubule chip and used FEM analysis to extract cellular OCR — an approach closely analogous to ours and a direct experimental complement to the simulations reported here. Integrating analogous sensors into the present PET- and PDMS-chip platforms is the highest-priority next step of this project and will allow direct quantitative validation of the FEM predictions.

### 3.2 Material-dependent reconstruction of I/R injury (PDMS chip vs. PET chip)

**FIGURE 3:**
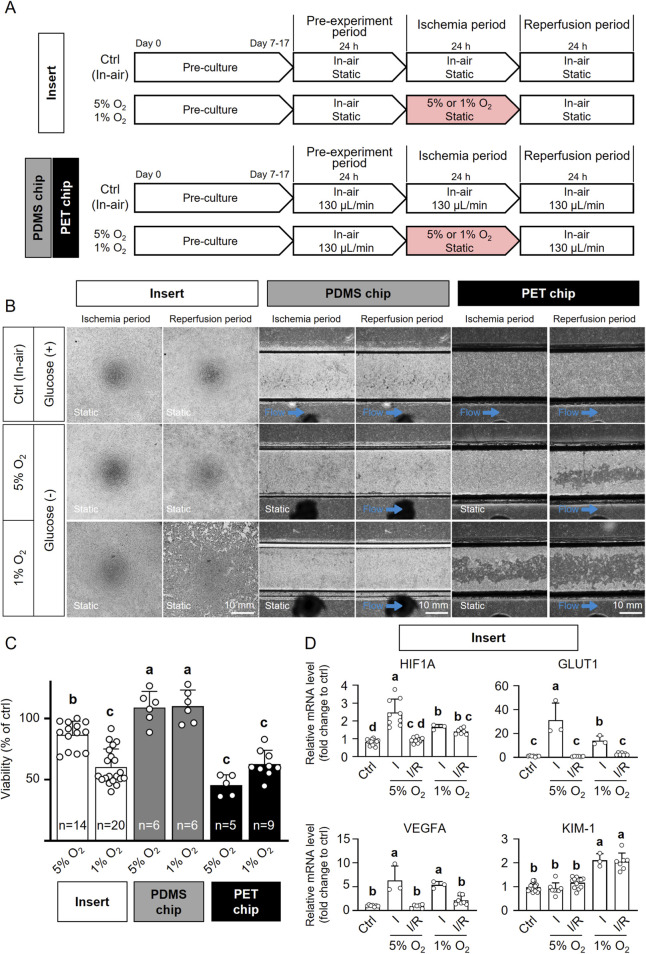
Cell morphology, viability, and gene expression in a renal ischemia–reperfusion model. **(A)** Experimental protocol for establishing the renal ischemia–reperfusion model. During the ischemic period, cells were cultured under static conditions without medium perfusion. **(B)** Representative images of cell morphology under in-air, 5% O_2_, and 1% O_2_ conditions. In each condition, the left images were acquired 24 h after the ischemia period, and the right images were acquired 24 h after the reperfusion period. **(C)** Cell viability relative to the control (in-air) was assessed using the Cell Counting Kit-8. White, gray, and black bars represent the culture insert, PDMS chip, and PET chip, respectively. Each dot represents one insert or chip. Data are presented as mean ± SD from at least three independent experiments. **(D)** Gene expression analysis by qPCR following ischemia–reperfusion using a cell culture insert. In **(C,D)**, different letters indicate statistically significant differences between groups (*p* < 0.05), as determined by Compact Letter Display. Each experiment was repeated at least three times. The dots in the graph represent the number of inserts or chips used in the experiment.

To experimentally probe the FEM predictions, *in vitro* renal I/R models were established using the PDMS and PET chips with identical geometries. The detailed experimental protocol is shown in Figure 3A.

Under control (in-air, perfusion) conditions, both PDMS and PET chips maintained a confluent monolayer with no apparent morphological differences (Figure 3B). However, clear differences were observed under low-oxygen conditions between the PDMS and PET chips. In the PDMS chip, no morphological changes were observed even after 24 h of low-oxygen environments at 5% and 1% O_2_, and the monolayer remained intact after reperfusion. Similarly, cell viability was not significantly reduced under these conditions (Figure 3C). In contrast, the PET chip exhibited pronounced I/R injury. While a confluent monolayer was maintained after a low-oxygen environment at 5% O_2_, significant cell detachment was observed at 1% O_2_ (Figure 3B). After the reperfusion period, cell detachment became evident even at 5% O_2_ and further expanded under 1% O_2_ conditions (Figure 3B). Quantitative viability analysis using the Cell Counting Kit-8 confirmed a substantial reduction (∼50%) under both 5% and 1% O_2_ I/R conditions (Figure 3C).

These experimental results are consistent with the FEM-predicted differences in oxygen availability and OCR (Figure 2B). Importantly, despite identical nominal oxygen conditions, only the PET chip reproduced ischemia-like injury, whereas the PDMS chip failed to induce detectable damage. This indicates that oxygen transport properties, rather than incubator oxygen levels alone, determine the ability to reproduce I/R injury. The absence of detectable injury in the PDMS chip, despite exposure to low-oxygen conditions, suggests that nominal oxygen levels do not necessarily reflect the actual oxygen environment around cells. This discrepancy can be explained by continuous oxygen diffusion through PDMS, which compensates for oxygen consumption by the cells. As a result, the cells were not exposed to oxygen-deprivation conditions sufficient to induce detectable ischemia-like injury under the present experimental setting. In contrast, the PET chip exhibited ischemia-like injury, indicating that oxygen supply was insufficient to meet cellular demand. These results highlight that the balance between oxygen supply and cellular consumption determines the establishment of ischemia, rather than the external oxygen setting alone.

In this study, PET and PDMS are used as two materials with different oxygen permeability but identical designs; however, other materials will also be considered in future studies. For example, cyclic olefin copolymer (COC), cyclic olefin polymer (COP), polymethyl methacrylate (PMMA), and polystyrene (PS) are potential alternative chip materials for I/R modeling. These materials combine low oxygen permeability with low molecular absorption and are suitable for mass production (van Midwoud et al., 2012). Polymethylpentene (PMP) is a material with high gas permeability; as a thermoplastic resin with high transparency and heat resistance, it is also used in cell culture applications (Nishikawa et al., 2022). Here, PDMS and PET were selected as examples of materials with significantly different gas permeability among commonly used MPS materials; however, since this method is also applicable to any pair of materials with known O_2_ transport properties as mentioned above, future research will allow for a certain degree of flexibility in material selection.

### 3.3 Comparison with conventional cell culture platform (insert vs. PET chip)

To position the PET chip relative to conventional *in vitro* models, I/R experiments were performed using a cell culture insert as the conventional platform. Under in-air conditions (control), the insert maintained a confluent monolayer similar to that observed in microfluidic chips (Figure 3B). After exposure to low-oxygen conditions (5% O_2_ and 1% O_2_) for 24 h, no apparent morphological changes were observed; however, after the reperfusion period, cell detachment occurred under 1% O_2_. In the cell viability assay, cell viability was slightly lower at 5% O_2_ than at ambient conditions, but it dropped sharply at 1% O_2_ (Figure 3C).

Compared with the PET chip, the insert exhibited milder injury, indicating less severe oxygen limitation. This observation is consistent with the FEM analysis, which predicted higher oxygen availability in the insert than in the PET chip under comparable conditions. The difference likely arises from diffusion-driven oxygen supply from the medium and the surrounding air in the insert system, whereas the PET chip restricts oxygen transport, resulting in a more pronounced oxygen deficit and, consequently, stronger cellular injury. These results suggest that although conventional cell culture insert systems can reproduce responses to low-oxygen concentrations, PET-based microfluidic systems enable the development of a more severe and controllable ischemic environment.

### 3.4 Biological characterization of hypoxia response in the insert platform

Under hypoxia, HIF-1α protein is stabilized and drives transcription of a coordinated pathway that includes the glucose transporter GLUT1 (metabolic adaptation by shifting toward glycolysis) and the angiogenic factor VEGFA. KIM-1, a type-I membrane protein expressed by injured proximal-tubule cells, is induced after sublethal or lethal tubular injury and is widely used as an early marker of acute kidney injury (Tanase et al., 2019). The expected pattern is therefore: HIF1A, GLUT1 and VEGFA induction under low oxygen conditions with attenuation upon reoxygenation, and KIM-1 induction specifically under conditions of cellular injury. A schematic of the relevant pathway is provided in Supplementary Figure S6. With this framework in mind, we examined gene expression in the insert system before and after reperfusion. HIF1A expression increased significantly under 5% and 1% O_2_ conditions (Figure 3D). HIF1A levels in the 5% O_2_ I/R group decreased after the reperfusion period following the ischemic period. GLUT1 and VEGFA are genes downstream of HIF-1α. These two genes exhibit expression patterns similar to HIF1A, and their upregulation was reversed after the reperfusion period following both the 5% and 1% O_2_ conditions. No significant changes in KIM-1, a marker of renal cell damage, were observed in the 5% O_2_ condition group compared to the in-air condition group (Ctrl). In contrast, KIM-1 expression was upregulated in the 1% O_2_ condition group after the ischemia period compared to the in-air condition group (Ctrl), and this upregulation was not reversed even after the reperfusion period.

HIF1A and its downstream genes, GLUT1 and VEGFA, showed increased expression in the 1% O_2_ and 5% O_2_ conditions after the ischemia period compared to the in-air condition group (Ctrl). The results suggest that low-oxygen stimulation triggered a cellular response to adapt to a low-oxygen environment. Under 5% O_2_ conditions, the expression levels of each gene were higher than under 1% O_2_ conditions. This may be because cellular damage was more severe under 1% O_2_, potentially leading to reduced HIF1A transcriptional activity compared with 5% O_2_. To understand the reason for reduced HIF1A expression in the 1% O_2_ group, it is helpful to refer to studies examining the relationship between HIF-1α expression and the lysine demethylase (KDM) family under chronic and intermittent hypoxic conditions (Martinez et al., 2022). Under chronic hypoxic conditions, the transcriptional activity of HIF-1 and the expression of the HIF-target genes KDM4B and KDM4C increase. However, despite the increase in protein levels, the amount of oxygen required for KDM activity is limited; consequently, KDM4A, KDM4B, and KDM4C remain largely inactive, leading to an increase in histone 3 lysine 9 trimethylation (H3K9me3) at the HIF1A gene locus and, ultimately, a decrease in the amount of HIF1A mRNA transcribed (Martinez et al., 2022). Meanwhile, under intermittent hypoxia, HIF-1α levels increase compared to normal oxygen conditions (in-air). Although the expression levels of KDM4B and KDM4C rose slightly, they did not reach the same levels as under chronic hypoxia. However, in contrast to chronic hypoxia, the activity of KDM4A, KDM4B, and KDM4C increases, resulting in higher levels of H3K9 demethylation at the HIF1A gene compared to both normal oxygen conditions and chronic hypoxic conditions. It is believed that this mechanism leads to increased production of HIF1A mRNA. Based on the interpretation that 1% O_2_ corresponds to chronic hypoxia and 5% O_2_ to intermittent hypoxia, the results of this study are deemed reasonable.

HIF1A levels decreased in the 5% O_2_ group following reperfusion after exposure to a low-oxygen condition. This change is thought to result from the recovery from hypoxia or low-oxygen condition following reoxygenation, and other studies have also demonstrated that HIF-1α levels decrease over time after I/R (Conde et al., 2012). Furthermore, it has been reported that HIF-1α induction during reperfusion plays a role in preventing inappropriate repair following renal I/R (Conde et al., 2012), making it a critical cellular response to ischemia. The slight decrease in HIF1A in the 1% O_2_ group is thought to be due to this cellular response. GLUT1 and VEGFA are downstream of the HIF-1α signaling pathway and exhibited expression patterns similar to those of HIF1A. The HIF-1α–VEGFA signaling pathway has been implicated in I/R injury in other organ contexts (Ni et al., 2022), and our results showed a similar expression pattern.

KIM-1 is a gene expressed in the proximal tubule that is induced by cellular injury or exposure to toxic substances (Tanase et al., 2019). Under 5% O_2_ condition, KIM-1 expression was at the same level as in the in-air group, suggesting that there was no or only minor cellular injury. In contrast, under 1% O_2_ condition, KIM-1 expression was elevated compared to the in-air group, suggesting that hypoxia-induced cellular injury occurred under this condition. Based on these findings, it is suggested that the 1% O_2_ condition reproduces the cellular impairment and response to ischemic or low-oxygen conditions observed *in vivo*.

It is important to note that, in the PET chip, severe cell damage due to low oxygen levels was observed under 1% O_2_ conditions; although gene expression analysis could not be performed due to poor RNA harvest, the differences in cell morphology and survival rates observed between the PET chip and the PDMS chip can be interpreted in light of the results of gene expression analysis using inserts and the FEM analysis. The PET chip, which exhibited pronounced cell injury, showed oxygen depletion levels comparable to or more severe than those in the insert under 1% O_2_ conditions, where hypoxia-responsive genes and injury markers were strongly induced. This suggests that similar or even enhanced hypoxia-induced cellular responses would occur in the PET chip. In contrast, the PDMS chip did not exhibit detectable cell injury despite exposure to low nominal oxygen conditions. Considering the FEM results indicating sustained oxygen availability and OCR in the PDMS chip, it is likely that hypoxia signaling pathways were insufficiently activated. These observations collectively support the notion that cellular responses in microfluidic systems are governed by the balance between oxygen supply and consumption, rather than by the nominal oxygen concentration alone. These findings highlight the importance of integrating computational prediction and experimental observation to interpret cellular function in MPSs.

### 3.5 Evaluation of drug response under controlled oxygen conditions

Finally, the applicability of the model for drug evaluation was examined using 2-cyano-3,12-dioxo-olean-1,9-dien-28-oic acid (CDDO). CDDO is a synthesized triterpenoid that potently activates Nuclear factor erythroid 2–related factor 2 (Nrf2), promotes its nuclear translocation (Kurosaki et al., 2022), and induces the expression of downstream antioxidant proteins such as NAD(P)H quinone dehydrogenase 1 (NQO1) and heme oxygenase 1 (HO-1) (Liby et al., 2005). Although this compound was originally developed for the prevention and treatment of inflammation and cancer (Suh et al., 1999; Konopleva et al., 2004), a significant increase in eGFR, a marker of kidney function, was observed in cancer patients, leading to its evaluation as a therapeutic agent for kidney disease. CDDO was chosen in this research because it is a potent activator of the Nrf2 antioxidant programme with documented renoprotective effects in rodent AKI and I/R models (Wu et al., 2014; Mathis and Cui, 2016; Nezu et al., 2017); its methylated derivative, bardoxolone methyl, has been evaluated in clinical trials in chronic and diabetic kidney disease (Pergola et al., 2011; de Zeeuw et al., 2013; Ng et al., 2025), providing direct translational relevance; and its well-documented biphasic concentration–response — cytoprotective at nanomolar concentrations through Nrf2 activation and cytotoxic at micromolar concentrations through pro-apoptotic and mitochondrial mechanisms (Borella et al., 2019; Gibellini et al., 2014) — offers an internally rigorous test of platform discrimination between protective and toxic regimes. Alternative agents commonly used in *in vitro* renal I/R or oxidative-stress models include N-acetylcysteine (Shimizu et al., 2005; Hernández-Cruz et al., 2024), superoxide-dismutase mimetics (e.g., MnTBAP), mitochondria-targeted antioxidants (MitoQ, SkQ1) and inhibitors of regulated necrosis (necrostatin-1, ferrostatin-1); CDDO was preferred here for the reasons given.

**FIGURE 4:**
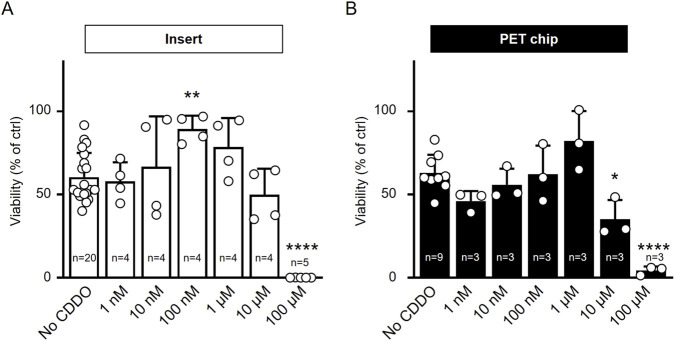
Evaluation of drug response under ischemia–reperfusion conditions. Cell viability in the insert **(A)** and PET chip **(B)** following treatment with CDDO. Cells were exposed to 1% O_2_ for 24 h to induce ischemia, followed by reperfusion under in-air conditions. Cell viability was assessed using Cell Counting Kit-8. Data are presented as the mean ± SD from at least three independent experiments. The dots in the graph represent the number of inserts or chips used in the experiment. Statistical significance was evaluated by one-way ANOVA followed by Dunnett’s test versus the No CDDO. *p < 0.05, **p < 0.01, ****p < 0.0001.

In this study, administration of CDDO showed a dose-dependent increase in cell viability up to approximately 100 nM in the insert, and at 100 nM, cell viability was significantly improved compared with the negative control (1% O_2_ I/R, No CDDO) group (Figure 4A). On the other hand, in the PET chip, a trend of dose-dependent increase in cell viability was observed up to a concentration of 1 μM (Figure 4B). However, at higher concentrations, cell viability decreased significantly at 100 µM in the culture insert and at concentrations exceeding 10 µM in the PET chip.

The renoprotective effects of CDDO are exerted by inducing increased expression of Nrf2, NQO1, and HO-1 in the kidneys, downregulating Kelch-like ECH-associated protein 1 (Keap1) expression, activating the Nrf2 signaling pathway, and increasing the expression of downstream target genes (Wu et al., 2014). It has also been shown to be effective in animal models of AKI and renal I/R by potently inhibiting the *de novo* synthesis of inflammatory enzymes such as inducible nitric oxide synthase and inducible cyclooxygenase-2 (Honda et al., 2000). At low concentrations (in the nanomolar range), CDDO protects cells and tissues from oxidative stress by enhancing the transcriptional activity of Nrf2 (Borella et al., 2019). Furthermore, at concentrations exceeding those required to activate the Nrf2 pathway (100 nM or higher), it can regulate the differentiation of various cell types, including tumor cell lines and primary cultured cells (Borella et al., 2019). On the other hand, at high concentrations (ranging from approximately 1 to 10 μM, depending on the molecular or cellular model), CDDO activates extrinsic apoptosis (Ito et al., 2001). In addition, it not only induces autophagy (Samudio et al., 2008) and inhibits telomerase activity (Deeb et al., 2010), but also, in a dose-dependent manner, causes an increase in hydrogen peroxide and superoxide anions within mitochondria. Consequently, this leads to increased carbonylation of mitochondrial proteins, resulting in mitochondrial depolarization, decreased mitochondrial mass, and morphological changes in the organelles, thereby inducing apoptosis (Gibellini et al., 2014). Our findings also suggest that a similar response occurs at high concentrations (10 μM or higher), resulting in reduced viability. In drug testing with CDDO in a renal I/R model, the previously reported cytoprotective effects at low concentrations and cell death and damage at high concentrations were generally reproduced, demonstrating the utility of this model for drug testing. Concerning the difference in the concentrations at which pharmacological effects were observed between the PET chip and the insert, results from morphological observations of the cells and FEM simulations suggest that the oxygen concentration around the cells was lower on the PET chip, and that the extent of damage the cells sustained prior to drug exposure was more severe on the PET chip. Therefore, this difference is thought to be due to the disparity in the damage sustained by the cells.

Taken together, these results support that drug responses in the *in vitro* renal I/R models are strongly influenced by the underlying oxygen microenvironment. Although CDDO exhibited both protective effects at low concentrations and cytotoxic effects at higher concentrations in both systems, the effective concentration range differed between the insert and the PET chip. This difference can be attributed to the severity of oxygen limitation, as the PET chip exhibits a more pronounced oxygen deficit, leading to greater baseline cellular injury and, consequently, reduced sensitivity to protective effects.

Importantly, these findings indicate that, even under identical nominal oxygen settings (1% O_2_), material-dependent differences in oxygen supply result in distinct cellular conditions and consequently alter the dose-response of CDDO. The broader hypothesis that drug efficacy is graded by the severity of oxygen limitation across multiple O_2_ levels—for example, that the protective concentration range shifts with O_2_ level—should be addressed in future work by performing CDDO dose-response analyses over a panel of oxygen conditions, such as 5% and 1% O_2_. Therefore, accurate evaluation of drug effects in MPSs requires precise control and understanding of oxygen availability at the cellular level. These results highlight that oxygen transport is a critical determinant not only of disease model fidelity but also of drug response, emphasizing the necessity of oxygen-controlled platforms for reliable *in vitro* pharmacological testing.

### 3.6 Limitations

This study has several limitations. First, microfluidic chips have lower throughput than conventional insert-based *in vitro* experimental models, limiting experimental scalability. This low throughput limits the experimental conditions and the number of experiments that can be conducted compared to experiments using conventional cell culture inserts. Therefore, improving throughput is a key challenge, and we are working to address it. Second, the complexity of perfusion systems, including tubing and pump configurations, may affect usability and reproducibility. In addition, the present study primarily focused on oxygen transport and cell viability, and only a limited set of gene expression markers was analyzed. A more comprehensive evaluation, including proteomic analysis and functional assays, will be needed to fully characterize cellular responses to ischemia-reperfusion. Third, although the FEM simulation was experimentally supported, further quantitative validation of the oxygen concentration using integrated sensors would strengthen the conclusions. Fourth, pharmacological evaluation with CDDO was performed at a single oxygen-deprivation level (1% O_2_). Although the platform-dependent shift in the protective concentration range is informative, a multi-oxygen dose-response (e.g., 5% O_2_ in addition to 1% O_2_) would be required to formally support the claim that drug response is graded by the severity of oxygen limitation. Finally, because the RPTEC/TERT1 cells used in this study are immortalized, their mitochondrial metabolic characteristics may differ slightly from those of primary cells. Additionally, there is a theoretical possibility that the sustained expression of hTERT may attenuate stress-induced aging responses. We believe these issues can be addressed by using primary cultured cells or cells derived from iPS organoids. Moreover, relevant cell types for future *in vitro* kidney I/R models include — beyond the proximal tubule epithelium used here — peritubular microvascular endothelial cells, podocytes, distal-tubule, and collecting-duct cells.

## 4 Conclusion

This study suggests that oxygen transport, rather than nominal oxygen conditions, is a critical determinant of the reproducibility of *in vitro* I/R models. By integrating FEM simulation with experimental observations, our results suggest that PDMS chips did not reproduce ischemia-like cellular injury under the present conditions due to their high gas permeability, whereas PET chips enable robust, controllable I/R-associated cellular responses by limiting oxygen supply at the cellular level. Importantly, the results highlight that cellular responses are governed by the balance between oxygen supply and consumption, rather than by the oxygen concentration set in the incubator. Even under identical nominal oxygen conditions, substantial differences in oxygen availability arise from material properties and culture configurations, leading to markedly different cellular phenotypes.

Furthermore, we suggest that the severity of oxygen limitation directly influences drug response. The differential effects of CDDO observed between the insert and PET chips indicate that pharmacological outcomes are highly dependent on the underlying oxygen microenvironment. These findings emphasize that accurate interpretation of drug efficacy requires careful consideration and control of oxygen transport conditions. Although it is difficult to directly compare oxygen concentrations *in vivo* and *in vitro*, reported tissue PO_2_ values in the human kidney, including approximately 50 mmHg in the cortex, ∼5–25 mmHg in the outer medulla, and <5 mmHg in ischemic tissue, provide useful contextual benchmarks for interpreting the severity of oxygen deprivation. In this context, our analysis indicates that the PET chip and the insert under reduced oxygen conditions generated strongly oxygen-deprived environments, whereas the PDMS chip exhibited a distinct oxygen transport profile characterized by relatively maintained oxygen availability and OCR despite the same nominal incubator oxygen settings. These findings suggest that the translational relevance of an *in vitro* I/R platform should not be judged by nominal oxygen concentration or a single dissolved oxygen value alone, but by the integrated oxygen transport state determined by material properties, culture configuration, perfusion, and cellular oxygen consumption. This study therefore provides a transport-based framework for selecting *in vitro* platforms whose cellular O_2_ environment can be tuned toward physiological or pathological targets. More broadly, our findings underscore the critical role of oxygen delivery in MPSs and highlight the need to integrate material selection, microfluidic chip design, and culture conditions, including medium composition and medium flow rate, to achieve biologically meaningful outcomes.

## Data Availability

The datasets presented in this study can be found in online repositories. The names of the repository/repositories and accession number(s) can be found in the article/Supplementary Material.
