# Supplementary material for: Material-dependent oxygen transport governs ischemia-reperfusion injury and drug response in a kidney microphysiological system
Source: Front Toxicol. 2026 Jul 10;8:1854389. doi: 10.3389/ftox.2026.1854389 (PMC13395555; doi:10.3389/ftox.2026.1854389)
Supplement: Supplementary file 1 [file Supplementaryfile1.docx]

**Figure S1**

**
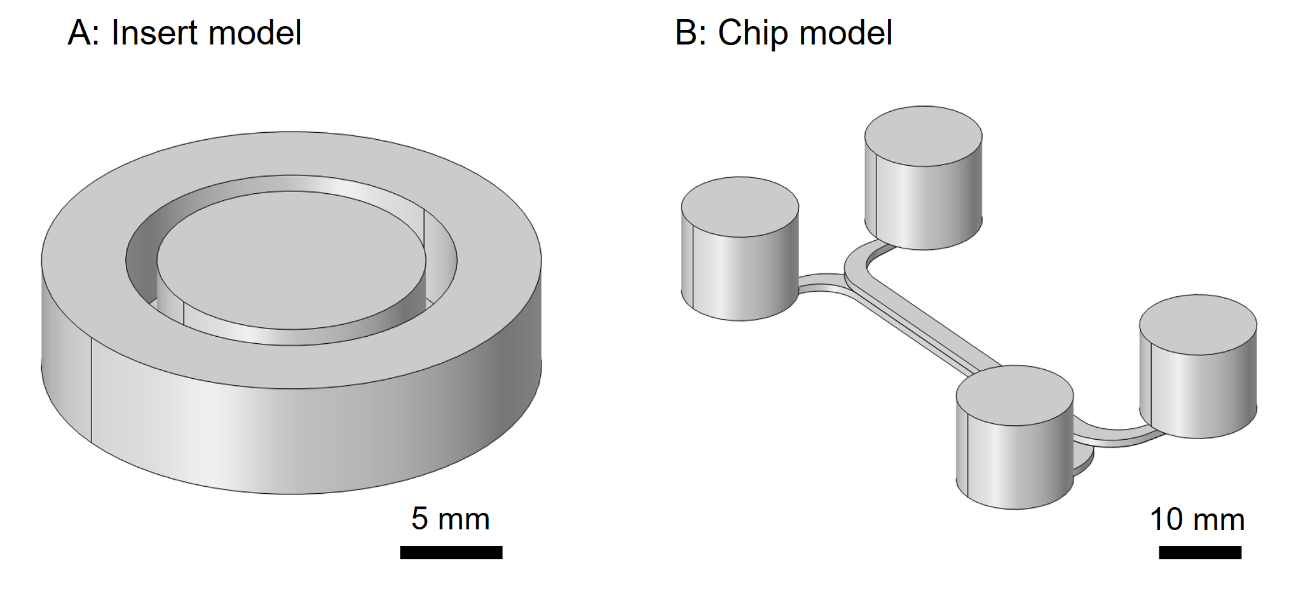
**

FIGURE S1. Geometry of FEM simulation models. (A) 12-well size culture insert model. (B) Double-layered microfluidic chip model. The culture medium, cell monolayer, and porous membranes were modeled in COMSOL Multiphysics.

**Figure S2**

**
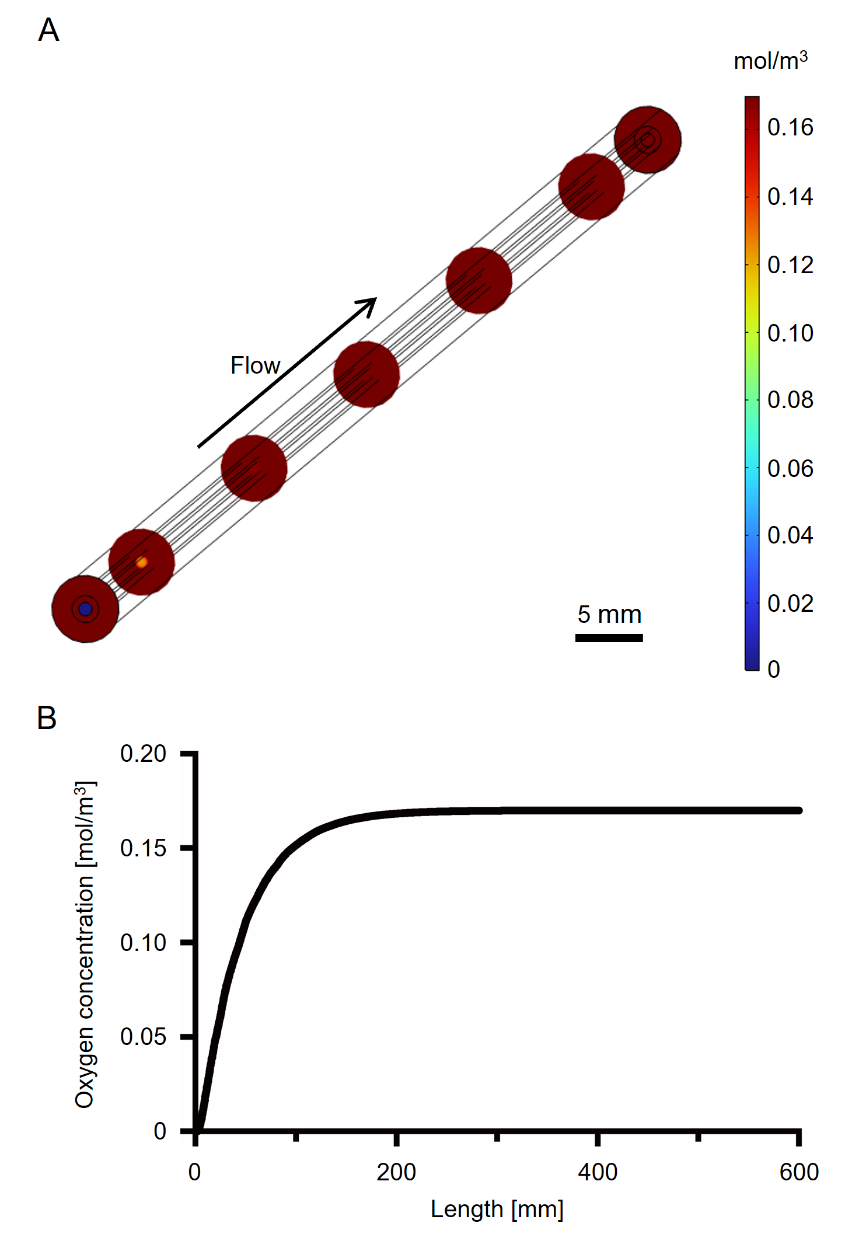
**

FIGURE S2. FEM simulation of oxygen dissolution into the culture medium through silicone tubing during perfusion. (A) Simulated change in oxygen concentration in the culture medium flowing through silicone tubing with an inner diameter of 1 mm and an outer diameter of 2 mm at a flow rate of 130 µL/min. (B) Oxygen concentration profile along the silicone tubing as culture medium with an initial oxygen concentration of 0 mol/m³ flows through at 130 µL/min.

**Table S1**

TABLE S1. Parameters for FEM simulation

| Parameter | Unit | Value | Reference |
| --- | --- | --- | --- |
| Diffusion coefficient | m^2^/s | 2.0 × 10^-9^ | Ref. 1 |
| Dissolved oxygen | mol/m^3^ | in-air: 0.179 | Ref. 2 |
|  |  | 5% oxygen: 0.048 | - |
|  |  | 1% oxygen: 0.010 | - |
| Basal OCR | mol/m^3^/s | 0.025 | Ref. 3 |
| Michaelis constant (*K_m_*) | mol/m^3^ | 0.001 | Ref. 4 |

**Table S2**

TABLE S2. Primer list for real-time PCR

| Gene | Forward primer (5′ to 3′) | Reverse primer (5′ to 3′) |
| --- | --- | --- |
| HPRT1 | CACCCTTTCCAAATCCTCAG | CTCCGTTATGGCGACCC |
| HIF1A | CAAGAACCTACTGCTAATGC | TTATGTATGTGGGTAGGAGATG |
| GLUT1 | TTGCAGGCTTCTCCAACTGGAC | CAGAACCAGGAGCACAGTGAAG |
| VEGFA | TTGCCTTGCTGCTCTACCTCCA | GATGGCAGTAGCTGCGCTGATA |
| KIM-1 | TGGCAGATTCTGTAGCTGGTT | AGAGAACATGAGCCTCTATTCCA |

**Figure S3**


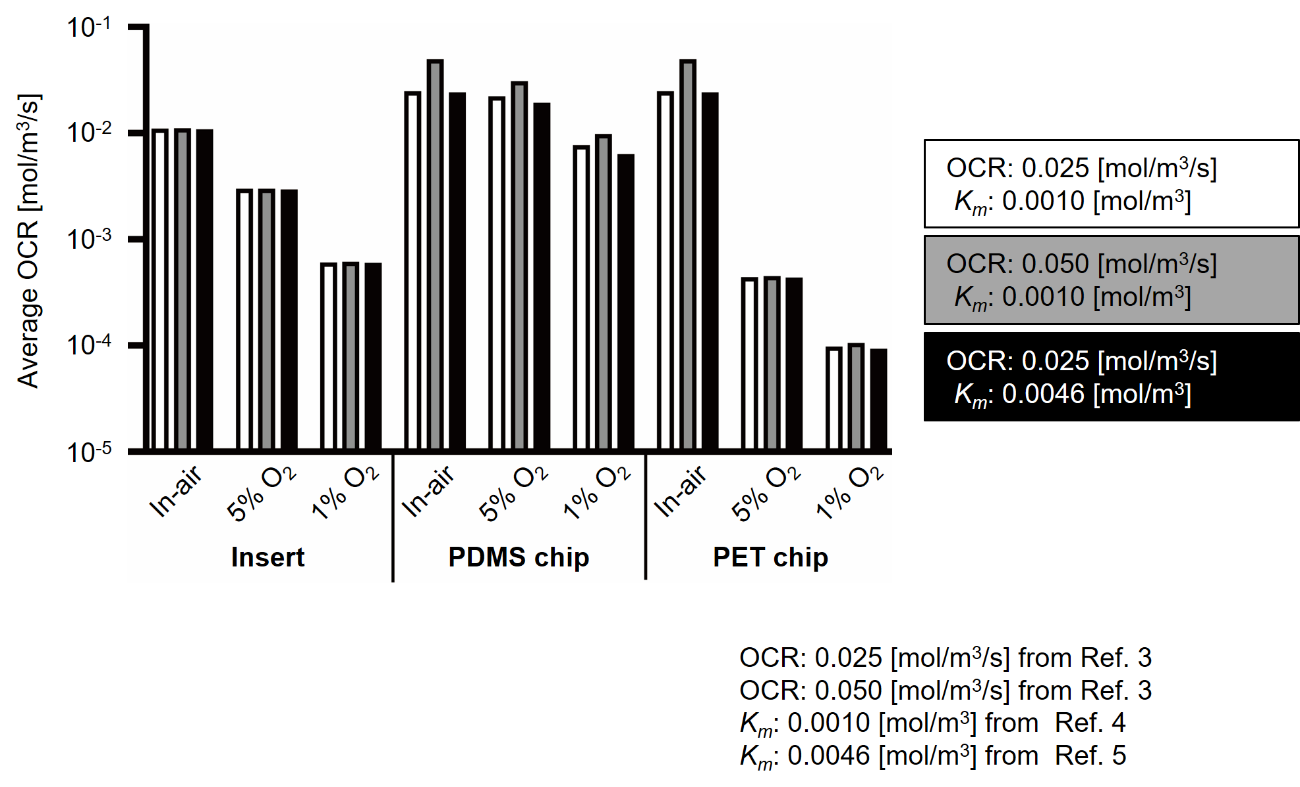


FIGURE S3. Sensitivity analysis of simulated average OCR by varying OCR and *K_m_*. Simulated 24 h-average OCR values obtained using different combinations of basal OCR and *K_m_*​ within the ranges reported in the literature. The average OCR values were nearly unchanged among the tested parameter sets, indicating that plausible variations in basal OCR and *K_m_*​ had little effect on the simulation results.

**Figure S4**

**
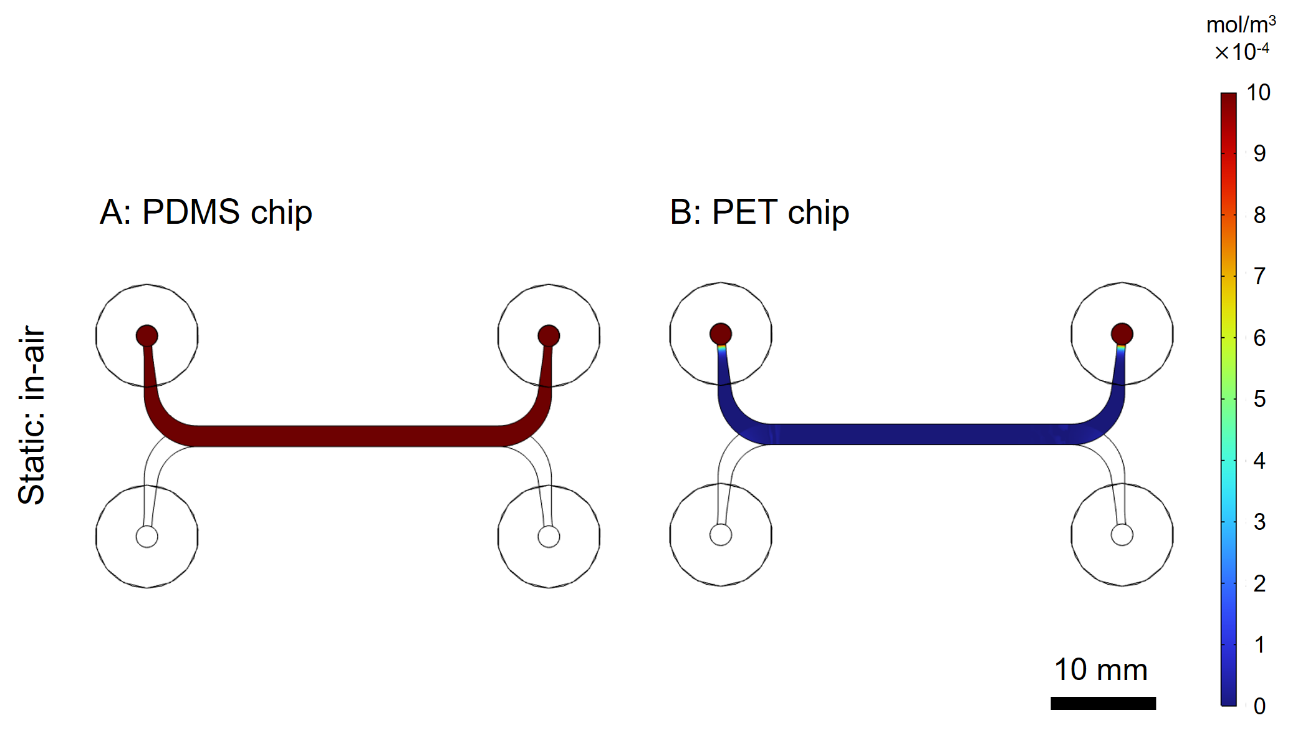
**

FIGURE S4. Oxygen concentration profiles in microfluidic chips under static (no-flow) conditions in the in-air environment. In the PDMS chip (A), oxygen levels were maintained due to diffusion through the gas-permeable material, whereas in the PET chip (B), oxygen was depleted within the channel in the absence of perfusion.

**Figure S5**

**
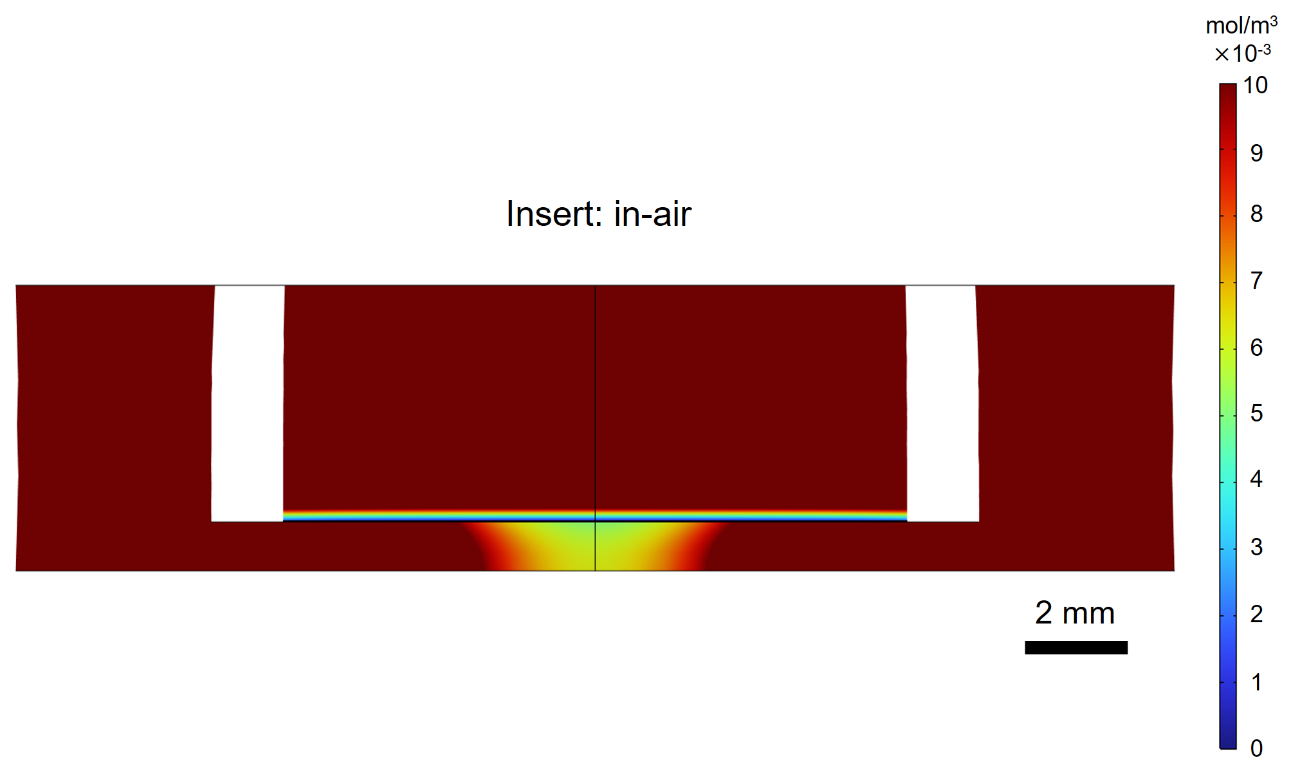
**

FIGURE S5. Cross-sectional simulation of oxygen concentration in the cell culture insert under static conditions in the in-air environment. Oxygen levels were lower toward the center of the culture area due to diffusion-limited transport through the culture medium and porous membrane, whereas higher oxygen levels at the peripheral region reflect oxygen supply from the basal chamber.

**Figure S6**


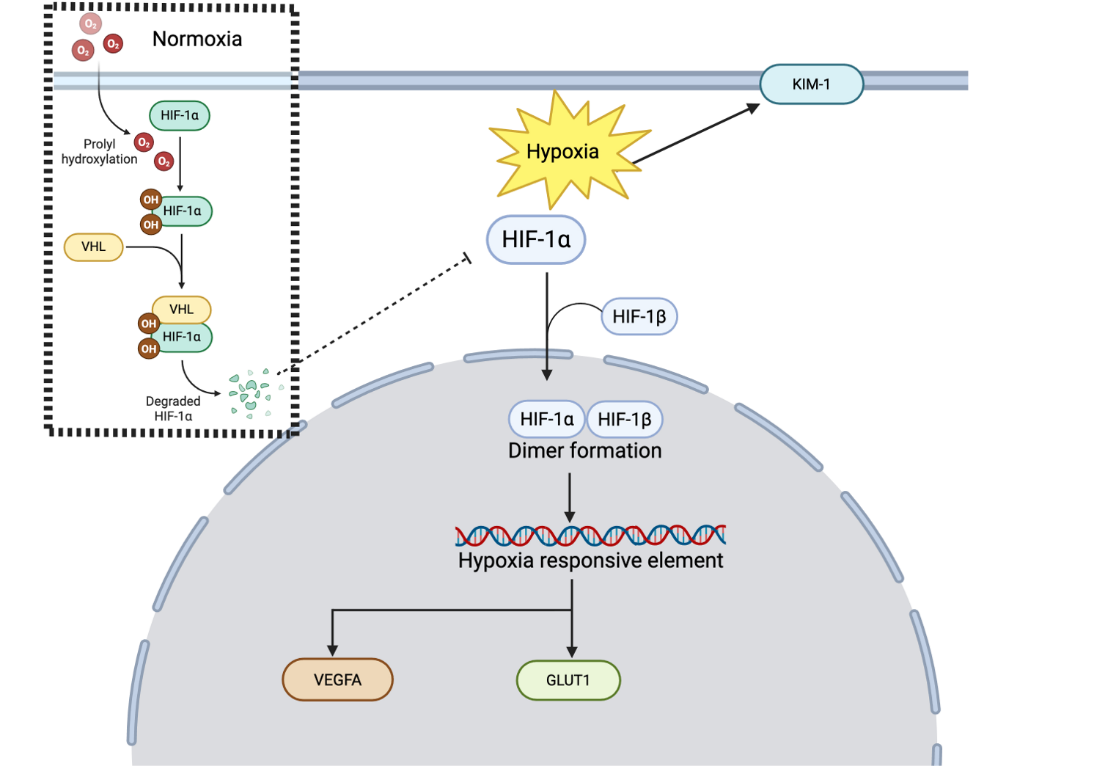


FIGURE S6. Schematic diagram illustrating the dynamics of the HIF-1α protein under normoxic and hypoxic conditions, the regulation of the expression of downstream genes GLUT1 and VEGFA, and the relationship with KIM-1.

**References**

1. XIAO, W., KODAMA, M., KOMORI, K. & SAKAI, Y. 2014. Oxygen-permeable membrane-based direct oxygenation remarkably enhances functions and gene expressions of rat hepatocytes in both 3D and sandwich cultures. *Biochemical engineering journal*, 91, 99.
2. AL-ANI, A., TOMS, D., KONDRO, D., THUNDATHIL, J., YU, Y. & UNGRIN, M. 2018. Oxygenation in cell culture: Critical parameters for reproducibility are routinely not reported. *PLoS One,* 13**,** e0204269.
3. SECKER, P. F., BENEKE, S., SCHLICHENMAIER, N., DELP, J., GUTBIER, S., LEIST, M. & DIETRICH, D. R. 2018. Canagliflozin mediated dual inhibition of mitochondrial glutamate dehydrogenase and complex I: an off-target adverse effect. *Cell Death & Disease,* 9**,** 226.
4. ROGERS, Z. J., COLOMBANI, T., KHAN, S., BHATT, K., NUKOVIC, A., ZHOU, G., WOOLSTON, B. M., TAYLOR, C. T., GILKES, D. M., SLAVOV, N. & BENCHERIF, S. A. 2024. Controlling Pericellular Oxygen Tension in Cell Culture Reveals Distinct Breast Cancer Responses to Low Oxygen Tensions. *Advanced Science,* 11**,** 2402557.
5. REFET-MOLLOF, E., NAJYB, O., CHERMAT, R., GLORY, A., LAFONTAINE, J., WONG, P. & GERVAIS, T. 2021. Hypoxic Jumbo Spheroids On-A-Chip (HOnAChip): Insights into Treatment Efficacy. Cancers (Basel), 13.
